# New Coxsackievirus B4 Genotype Circulating in Inner Mongolia Autonomous Region, China

**DOI:** 10.1371/journal.pone.0090379

**Published:** 2014-03-03

**Authors:** Xiaoling Tian, Yong Zhang, Suyi Gu, Yaochun Fan, Qiang Sun, Bo Zhang, Shaohong Yan, Wenbo Xu, Xueen Ma, Wenrui Wang

**Affiliations:** 1 Inner Mongolia Center for Disease Control and Prevention, Hohhot City, Inner Mongolia Autonomous Region, People's Republic of China; 2 WHO WPRO Regional Polio Reference Laboratory and Ministry of Health Key Laboratory for Medical Virology, National Institute for Viral Disease Control and Prevention, Chinese Center for Disease Control and Prevention, Beijing, People's Republic of China; University of Illinois at Chicago, United States of America

## Abstract

Hand, foot, and mouth disease (HFMD) surveillance was initiated in the Inner Mongolia Autonomous Region of China in 2007, a crucial scrutiny for monitoring the prevalence of enterovirus serotypes associated with HFMD patients. However, this surveillance mostly focused on enterovirus 71 (EV-A71) and coxsackievirus A16; therefore, information on other enterovirus serotypes is limited. To identify the other circulating enterovirus serotypes in the HFMD outbreaks in Inner Mongolia in 2010, clinical samples from HFMD patients were investigated. Six coxsackievirus B4 (CVB4) strains were isolated and phylogenetic analyses of *VP1* sequences were performed. Full-length genome sequences of two representative CVB4 isolates were acquired and similarity plot and bootscanning analyses were performed. The phylogenetic dendrogram indicated that all CVB4 strains could be divided into 5 genotypes (Genotypes I–V) with high bootstrap support (90–100%). The CVB4 prototype strain (JVB) was the sole member of genotype I. CVB4 strains belonging to genotype II, which were once common in Europe and the Americas, seemingly disappeared and gave way to genotype III and IV strains, which appear to be the dominant circulating strains in the world. All Chinese CVB4 strains belonged to Genotype V, a newly identified genotype supported by a high bootstrap value (100%), and are circulating only in mainland of China. Intertypic recombination occurred in the Chinese CVB4 strains with novel unknown serotype *EV-B* donor sequences. Two Chinese CVB4 strains had a virulent residue at position 129 of VP1, and one strain also had a virulent residue at position 16 of VP4. Increased surveillance is needed to monitor the emergence of new genetic lineages of enteroviruses in areas that are often associated with large-scale outbreaks. In addition, continued monitoring of enteroviruses by clinical surveillance and genetic characterization should be enhanced.

## Introduction

Human enteroviruses (EVs) are members of the genus *Enterovirus* within the family *Picornaviridae*, order *Picornavirales*, and consist of four species: *EV-A*, *EV-B*, *EV-C*, and *EV-D* [1]. Coxsackievirus B4 (CVB4) belongs to species *EV-B*, which currently consists of 61 serotypes, including echovirus (serotypes 1–7, 9, 11–21, 24–27, and 29–33), coxsackievirus group A (CVA, serotypes 9), coxsackievirus group B (CVB, serotypes 1–6), recently identified EV serotypes designated as EV-B69, EV-B73–B75, EV-B77–B88, EV-B93, EV-B97–B98, EV-B100–B101, EV-B106–B107, and EV-B110–B111, and the simian enterovirus SA5 [2]–[5]. *EV-B* viruses are common causes of human disease and are associated with a wide range of clinical manifestations, e.g., acute aseptic meningitis, acute myocarditis, acute flaccid paralysis, pancreatitis, hand, foot, and mouth disease (HFMD), and death in neonates, and even the same kind of disease outbreaks are usually associated with changes in different EV serotypes [6]–[9].

HFMD is a common contagious disease among children that occurs worldwide sporadically and in epidemics. HFMD typically occurs as outbreaks and is characterized by mucocutaneous papulovesicular lesions on the hands, feet, mouth, and buttocks. In the past several years, large outbreaks of HFMD occurred repeatedly in mainland of China, each involving more than 1,000,000 cases, and an increasing number of neurologic symptoms and deaths have been reported. HFMD has therefore become a significant public health issue in mainland of China [10], [11]. A large number of EV serotypes are associated with HFMD, with human enterovirus 71 (EV-A71) and CVA16 being the two major causative agents; however, the association of HFMD with other pathogens such as CVB4 infection is reported rarely [11]–[14].

HFMD surveillance was set up in the Inner Mongolia Autonomous Region in 2007, and it is a part of an EV virological surveillance system that is crucial for monitoring the prevalence of EV serotypes associated with HFMD patients. However, this surveillance was mostly focused on EV-A71 and CVA16; therefore, information on the pathogenic role of other EVs, their geographic distribution, and epidemiological profiles is still lacking. In this study, new genotype CVB4 strains were identified from HFMD patients and their genetic features were analyzed.

## Materials and Methods

### Viruses

This study did not involve human participants or human experimentation; the only human materials used were throat or rectal swabs collected from HFMD patients at the instigation of the Ministry of Health, People's Republic of China, for public health purposes. Written informed consent for the use of their clinical samples was obtained from all patients involved in this study. This study was approved by the second session of the Ethics Review Committee of the National Institute for Viral Disease Control and Prevention, Chinese Center for Disease Control and Prevention.

The CVB4 strains used in this study were isolated from throat swabs or rectal swabs collected from HFMD patients in five cities (Hohhot, Ordos, Baotou, Bayannur, and Hulunbeier) in the Inner Mongolia Autonomous Region of China. Viruses were isolated from original clinical specimens by propagation in human rhabdomyosarcoma (RD) and human larynx carcinoma (HEp-2) cells by conventional methods [15].

### Determination of the entire *VP1* nucleotide sequence of the CVB4 strains

Viral RNA was extracted from the viral isolates using a QIAamp Viral RNA Mini Kit (QIAGEN, Valencia, CA, USA). The entire *VP1* region of the CVB4 strains was amplified by reverse transcription-polymerase chain reaction (RT-PCR) with in-house primers that flanked the *VP1* region: CVB4-VP1-2397Y: 5′-GTACGCATGTTGAGGGACAC-3′ (nucleotides 2397–2416, relative to strain CVB4/JVB), and CVB4-VP1-3468Z: 5′-TTGTGCATTGGCATCTGGC-3′ (nucleotides 3449–3468, relative to strain CVB4/JVB). RT-PCR was performed using an Access RT-PCR Kit (Promega, USA) according to the manufacturer instructions. The PCR products obtained were purified using a QIAquick Gel Extraction Kit (QIAGEN), and the amplicons were sequenced bidirectionally using an ABI PRISM 3130 Genetic Analyzer (Applied Biosystems, Hitachi, Japan).

### Full-length genome sequencing of two CVB4 representative strains

Initially, viral RNA was converted to cDNA using a random priming strategy, and the complete genome sequences of the viruses were then acquired according to the published strategies for EV sequencing [3]. Briefly, overlapping fragments representing the complete genomes were amplified by RT-PCR using the specific, non-degenerate primers listed in Table 1, and a primer-walking strategy was used to close the gaps as necessary. The PCR products obtained were purified for sequencing using a QIAquick Gel Extraction Kit (QIAGEN), and the amplicons were then sequenced bidirectionally using fluorescent dideoxy-chain terminators and an ABI PRISM 3130 Genetic Analyzer (Applied Biosystems, Foster City, CA, USA). The 5′-segment sequences were determined using a 5′-Rapid Amplification of cDNA Ends Core Set (Takara Biomedicals, Dalian, China), according to the manufacturer's instructions.

**Table 1:** PCR and sequencing primers.

Primer	Nucleotide position (nt)	Primer sequence (5′–3′)	Orientation	Reference
0001S48		GGGGACAAGTTTGTACAAAAAAGCAGGCTTTAAAACAGCTCTGGGGTT	Forward	[33]
CVB4-1A	1218–1238	GCATATTCTGCCCAAAGAGCCC	Reverse	This study
CVB4-2S	1078–1098	TCAGTGATGAAGAGGCGACA	Forward	This study
CVB4-2A	1458–1478	ATACCTGCATTGCACACAGC	Reverse	This study
CVB4-3S	1333–1353	TGAGGCAGAAATGGGGTGTA	Forward	This study
CVB4-3A	2576–2596	GTCTGCATCGTGTCACTTGG	Reverse	This study
CVB4-VP1-2397Y	2397–2416	GTACGCATGTTGAGGGACAC	Forward	This study
CVB4-VP1-3468Z	3449–3468	TTGTGCATTGGCATCTGGC	Reverse	This study
CVB4-5S	2998–3018	AGTACCAGCAGCCAAGGAGA	Forward	This study
CVB4-5A	3797–3818	GGTGGGAGTTTCAGGAATGA	Reverse	This study
CVB4-6S	3674–3694	AGGGAATTGTTGGCTTTGCT	Forward	This study
CVB4-6A	4587–4609	CAGAACAAAGACACGTCCTTGC	Reverse	This study
CVB4-7S	4388–4409	TGCCGTATTGAACCTGTATGC	Forward	This study
CVB4-7A	5677–5695	CATGTTTGGGAATTTGCT	Reverse	This study
CVB4-8S	5422–5442	ACGGCGAGTTTACAATGCTT	Forward	This study
CVB4-8A	6326–6345	TGTCCATACATTCCTTCAA	Reverse	This study
CVB4-9S	6328–6349	GAAGGAATGTATGGACAAGTA	Forward	This study
7500A^a^		GGGGACCACTTTGTACAAGAAAGCTGGG(T)_24_	Reverse	[33]

### Phylogenetic analysis

The nucleotide and deduced amino acid sequences of the CVB4 isolates were compared to the prototype strain JVB and other *EV-B* serotype prototypes by pairwise alignment using the MEGA program (version 5.0; Sudhir Kumar, Arizona State University, Tempe, AZ, USA) [16]. Phylogenetic trees were constructed by the neighbor-joining method implemented in the MEGA program using the Kimura-2-parameters model. Regions containing alignment gaps were omitted from the analysis. The branch lengths of the dendrogram were determined from the topologies of the trees and were obtained by majority rule consensus among 1000 bootstrap replicates. Bootstrap values greater than 80% were considered statistically significant for grouping.

### Recombination analysis

Nucleotide alignment of the genome sequences of an Inner Mongolia CVB4 strain (HHHT34T) and *EV-B* prototype strains was generated using the MEGA program (version 5.0; Sudhir Kumar, Arizona State University, Tempe, AZ, USA) [16]. Once aligned, similarity plot and bootscanning analyses were performed using the Simplot program (version 3.5.1; Stuart Ray, Johns Hopkins University, Baltimore, MD, USA) [17].

### Nucleotide sequence accession numbers

The complete *VP1* nucleotide sequences (852 nucleotides) of the CVB4 strains that were determined in this study have been deposited in GenBank under the accession numbers KF781519 to KF781523. The whole genome sequences of the two CVB4 strains that were determined in this study have been deposited in GenBank under the accession numbers KF781524 and KF781525.

## Results

### New CVB4 genotype circulating in mainland of China

Complete *VP1* sequences were used to describe the phylogenetic relationship between CVB4 strains by defining the different genotypes of these viruses. To determine the molecular epidemiology of Chinese CVB4 strains associated with HFMD epidemics, a phylogenetic dendrogram was constructed with nine selected Chinese CVB4 strains isolated from clinical specimens of HFMD patients (six CVB4 strains isolated from Inner Mongolia in 2010, one from Inner Mongolia in 2007, one from Guangxi in 2010 [6], and another from Jiangsu in 2010) and 79 international CVB4 strains that were selected randomly on the basis of their genetic relationships.

**Figure 1:**
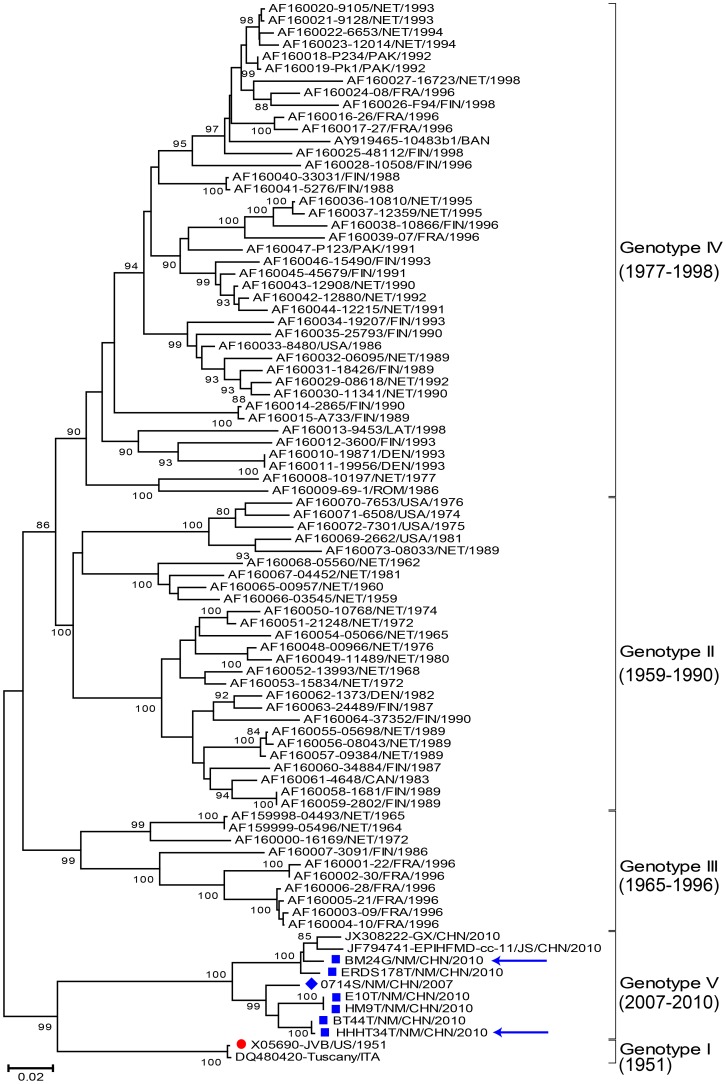
Phylogenetic analyses of the six Inner Mongolia CVB4 strains and reference strains from GenBank using the 852-bp *VP1* region sequence. The strains indicated by blue squares are the CVB4 strains isolated in this study; the strain indicated by a red circle is the prototype CVB4 strain. The arrows indicate the strains in which whole genome sequencing was performed.

The phylogenetic dendrogram indicated that all CVB4 strains could be divided into 5 genotypes (Genotypes I–V) with high bootstrap support (90–100%), and all Chinese CVB4 strains clustered into a newly identified genotype, Genotype V, which was supported by a high bootstrap value (100%) (Figure 1). The prototype JVB strain differed from the other strains by 13.7–17.8% and clustered separately from all of the CVB4 strains that were obtained in this study or were downloaded from GenBank database; accordingly, it was reconfirmed as the sole member of genotype I. All other CVB4 strains, including all Chinese CVB4 strains, were clearly assigned to Genotypes II–V, and this finding was based on the fact that the mean nucleotide variation within the genotypes ranged from 5.18% (within Genotype V) to 9.87% (within Genotype II), and the mean nucleotide variation between the genotypes ranged from 14.58% (between Genotypes I and V) to 19.03% (between Genotypes IV and V), which conformed to the generally accepted criterion for EV genotype demarcation (15% nucleotide variation in the *VP1* region between EV genotypes).

### Full-length genomic characterization of Inner Mongolia CVB4 strains

On the basis of *VP1* region nucleotide divergence, two representative Inner Mongolia CVB4 strains, HHHT34T/NM/CHN/2010 (hereafter referred to as HHHT34T) and BM24T/NM/CHN/2010 (hereafter referred to as BM24T), were selected, and their full-length genome sequences were determined. The results showed that they were similar to the prototype strain, strain JVB/USA/1951, with 7,393–7,394 nucleotides (strain BM24T having a single-nucleotide insertion in its 3′-untranslated [UTR] region compared with the prototype strain), including a 5′-UTR of 741 nucleotides (two nucleotide deletions compared with the prototype strain), a single open reading frame of 6,552 nucleotides encoding a single polyprotein of 2,183 amino acids, and a 3′-UTR of 100–101 nucleotides preceding the poly(A) tract. These sequences shared 86.8% nucleotide sequence identity with each other, indicating the co-circulation of different sub-genotypes of CVB4 in Inner Mongolia.

A comprehensive comparison of the nucleotide sequences and deduced amino acid sequence identities between the two Inner Mongolia CVB4 strains and the prototype strain of *EV-B* is shown in Table 2. The nucleotide sequence identities between these 2 CVB4 strains and the prototype CVB4 strain were 85.0–85.1%, 80.6–81.6%, and 79.8–82.6% in the *P1*, *P2*, and *P3* coding regions, respectively. The deduced amino acid sequence identities between these two CVB4 strains and the prototype CVB4 strain were 98.3–99.0%, 95.8%–96.1, and 94.3–95.5% in the P1, P2, and P3 coding regions, respectively (Table 2). The results indicated that in the capsid region, the two Inner Mongolia CVB4 isolates were closer to the CVB4 prototype strain than to the other *EV-B* viruses, but in the non-capsid regions (*P2* and *P3* regions), they were both close to the other *EV-B* prototype strains, indicating that these two Inner Mongolia CVB4 isolates were recombinants.

**Table 2:** Pairwise nucleotide and amino acid sequence identities between the CVB4 strains and prototype EV-B strains.

	HHHT34T/NM/CHN/2010		BM24T/NM/CHN/2010	
	Nucleotide identity (%) (Amino acid identity [%])		Nucleotide identity (%) (Amino acid identity [%])	
	CVB4 prototype	EV-B prototypes	CVB4 prototype	EV-B prototypes
*5*′*-UTR*	82.7	79.3–88.5	82.3	78.9–88.0
*VP4*	79.7 (97.1)	67.6–81.1 (76.8–98.5)	83.0 (98.5)	66.6–81.6 (76.8–98.5)
*VP2*	86.3 (99.6)	65.9–73.2 (73.6–83.9)	86.8 (98.8)	65.7–72.6 (73.6–83.5)
*VP3*	84.3 (99.1)	62.3–73.1 (66.5–84.0)	84.3 (98.7)	61.8–72.2 (66.1–84.0)
*VP1*	85.5 (98.5)	54.4–65.1 (55.9–73.6)	85.4 (97.8)	54.8–66.0 (55.9–73.6)
*2A*	76.4 (90.6)	71.6–79.1 (80.6–92.6)	79.1 (91.3)	72.9–78.9 (81.3–92.0)
*2B*	77.7 (96.9)	74.7–85.1 (92.9–97.9)	79.1 (96.9)	74.4–83.5 (92.9–97.9)
*2C*	85.3 (98.1)	79.5–84.8 (96.6–98.7)	82.0 (98.4)	79.0–82.9 (96.9–99.3)
*3A*	82.0 (87.6)	74.5–84.2 (91.0–96.6)	78.6 (87.6)	73.7–85.0 (89.8–95.5)
*3B*	80.3 (95.4)	71.2–86.3 (86.3–95.4)	84.8 (100.0)	71.2–89.3 (90.9–100.0)
*3C*	82.5 (96.7)	76.6–85.9 (95.6–98.9)	79.5 (93.4)	74.4–85.6 (92.3–96.7)
*3D*	79.2 (95.8)	78.1–86.9 (94.8–98.4)	82.6 (96.3)	78.1–82.9 (93.9–96.7)
*3*′*-UTR*	90.2	78.8–92.2	87.5	76.9–94.2

Two amino acid residues in the capsid protein were previously proved to be associated with the virulence of CVB4: one is a serine residue at position 16 of VP4 [18], and the other is a methionine residue at position 129 of VP1 [19]. Both sequences have a methionine residue (virulent) at position 129 of VP1. At position 16 of VP4, strain HHHT34T has a serine residue (virulent), while strain BM24T has an asparagine residue.

### Both strains recombined with different unknown serotype *EV-B* donors

**Figure 2:**
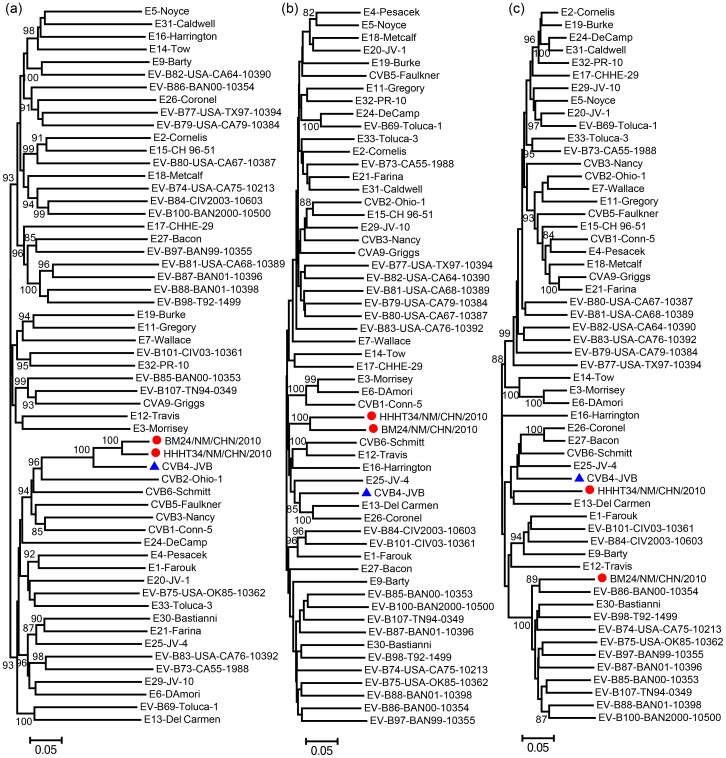
Unrooted trees representing the phylogenetic relationships among Inner Mongolia CVB4 strains and other *EV-B* strains. The phylogenetic trees based on the nucleotide sequences for the *P1* (a), *P2* (b), and *P3* coding sequences (c) were constructed from nucleotide sequence alignment using the neighbor-joining algorithm of MEGA 5.0 software. The numbers at the nodes indicate bootstrap support for that node (percent of 1000 bootstrap pseudoreplicates). The scale bars represent the genetic distance, and all unrooted trees have the same scale.

Alignments of the *P1*, *P2*, and *P3* region nucleotide sequences were carried out among the two selected Inner Mongolia CVB4 strains described above and the prototype *EV-B* strains, and phylogenetic trees were constructed (Figure 2). The phylogenetic dendrogram suggested that the Inner Mongolia CVB4 strains are monophyletic in the *P1* region, and both CVB4 strains clustered together with the CVB4 prototype strain (Figure 2a), confirming the classification of these isolates as a single enterovirus serotype. Interestingly, in the non-capsid *P2* region, these 2 CVB4 isolates had the highest nucleotide identity (85.1%) with each other and did not cluster with the CVB4 prototype strain (Figure 2b), while in the non-capsid *P3* region, HHHT34T and BM24T had the highest nucleotide identity (83.1% and 85.5%) with the echovirus 13 and EV-B86 prototype strains, respectively (Figure 2c).

**Figure 3:**
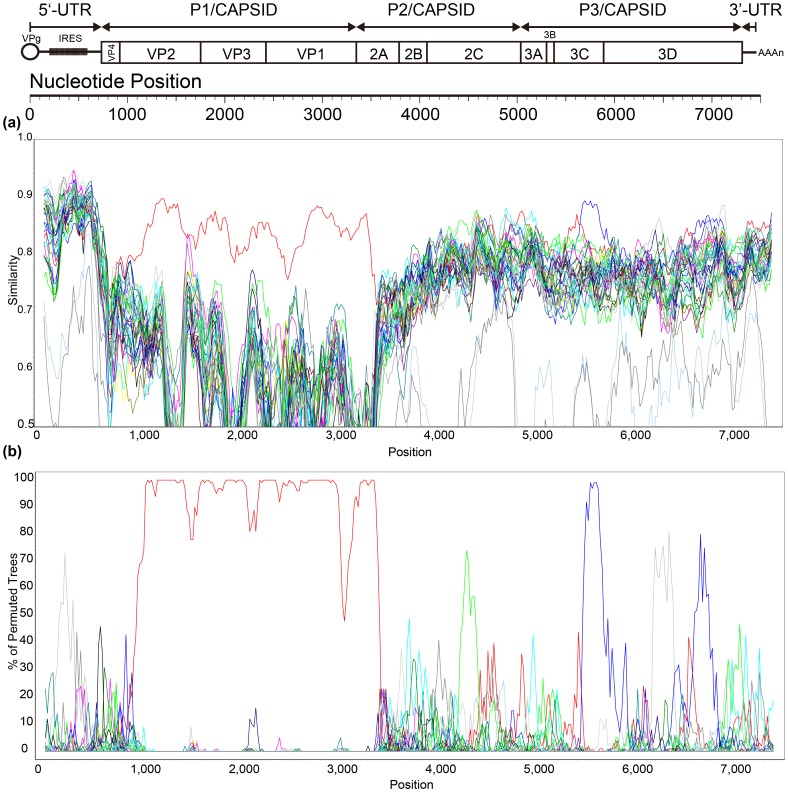
Similarity plot and bootscanning analyses of the whole genome of the Inner Mongolia CVB4 strains. Gene structure organization (a), similarity plot (b), and bootscanning analysis (c) of complete EV-B genomes using a sliding window of 200 nt moving in 20-nt steps. The HHHT34T/NM/CHN/2010 isolate was used as a query sequence and is indicated in the lower right corner. For each bootscanning analysis, the names of the viruses used as the query sequence are indicated in the upper right corner.

Similarity plot and bootscanning analyses with strain HHHT34T (selected as a representative strain) also revealed recombination between the Inner Mongolia CVB4 strains and other *EV-B* strains (Figure 3). On the basis of the above genetic characterization of the two CVB4 isolates, it can be concluded that recombination events occurred in the non-capsid region and these two CVB4 isolates had co-circulated with an unknown serotype *EV-B*. Considering that they were isolated from different regions (Hohhot City and Bayannur City, respectively) of Inner Mongolia, it may be suggested that the two CVB4 isolates recombined with different *EV-B* sequences or experienced different transmission patterns.

## Discussion

The protein encoded by the *VP1* gene plays an important role in characterizing antigenicity [20], and analysis of the complete *VP1* region sequence, which is considered the most reliable and rigorous method for determining EV genotypes, provides the most useful information for molecular epidemiology studies [21], [22]. Phylogenetic analysis of complete *VP1* sequences has identified five CVB4 genotypes (Genotypes I–V), and the overall pattern of strain segregation in the phylogenetic tree supported this criterion for CVB4 genotyping. The CVB4 prototype strain (JVB) is the sole member of Genotype I. Our study also showed that CVB4 strains belonging to Genotype II, which were once transmitted in Europe (Netherlands, Finland, and Denmark) and North America (USA and Canada), seemingly disappeared and gave way to Genotype III and IV strains, which appear to be the dominant circulating strains in the world and this may also indicate the previous transmission of this genotype. Phylogenetic analysis, which was supported by high bootstrap values, indicated that the Chinese CVB4 strains belong to Genotype V, a newly identified genotype that emerged and is circulating only in mainland of China.

Previous molecular epidemiology studies of CVB4 by Chu et al. [23] and Mulders et al. [24] showed that CVB4 strains could be divided into different clusters based on the 3′-*VP1* region (extrapolated genotype demarcation was 6.2%) [23], *VP1*/*2A* junction region (extrapolated genotype demarcation was 15%) [24], and entire *VP1* region (extrapolated genotype demarcation was 11%) [24]. However, the different genotypes reported in these studies correspond to Genotypes I–IV in our study, in which a difference of at least 15% in the complete *VP1* nucleotide sequence of the CVB4 strains was used to distinguish genotypes.

Recombination is a well-known phenomenon in EVs, and may play an important role in the replication cycle, transmissibility, and pathogenicity of EVs [25], [26]. In this study, genetic characterization of the full-length genomes of two CVB4 isolates, HHHT34T and BM24T, was performed. Interestingly, strains HHHT34T and BM24T recombined with different unknown serotype *EV-B* donor sequences, which was evident by the fact that these CVB4 isolates had the highest nucleotide identity in the non-capsid *P2* region, but in the non-capsid *P3* region, HHHT34T had the highest nucleotide identity with the echovirus 13 prototype strain, and BM24T had the highest nucleotide identity with the EV-B86 prototype strain.

Since 1995, EV virological surveillance was set up and gradually strengthened in the Inner Mongolia Autonomous Region. The surveillance program aims at poliovirus eradication by identifying important strains (wild type as well as vaccine-derived polioviruses), identifying EV outbreaks (especially HFMD epidemics), estimating the circulation patterns of different EV serotypes in the human community, and controlling possible circulation pathways of these viruses. In this study, we identified six CVB4 strains from five regions of Inner Mongolia, which were isolated in 2010. Although the number of CVB4 isolates was limited, it showed that CVB4 was widely distributed and transmitted in this region, which was also further evident by the fact that strain HHHT34T had high divergence with strain BM24T.

HFMD is caused by acute EVs infections, particularly by EVs belonging to the *EV-A* species, and the most common causative pathogens within *EV-A* are EV-A71 and CVA16, which are the 2 major causative agents of HFMD [11], [27]. Some other *EV-A*, such as CVA6 and CVA10, were reported causing HFMD outbreaks frequently in recent years [14], [28]–[30]. Beside these, some EVs belonging to the *EV-B* species were occasionally reported causing HFMD, including some echoviruses [9], [31] and CVBs [6], [14], [32].

In the patients with positive CVB4 isolates in this study, the median age at the time of CVB4 infection was four years (range, 2–11 years), which is slightly larger than in patients with EV-A71 and CVA16 infections [10]. In all of the patients affected by CVB4, mucocutaneous papulovesicular lesions were present on their hands, feet, mouth, and buttocks, which are the typical symptoms of HFMD, and which resolved spontaneously. This pathogenicity is consistent with the possible virulence of the two Chinese CVB4 strains. However, no serum samples were collected from the patients during the case investigation unfortunately, so it is a pity lack of serological evidence to confirm these cases infected with CVB4, and a possible association between CVB4 infection and HFMD requires a further detailed, controlled study.

China has been one of the countries that are most seriously affected by HFMD epidemics since 2007 [10], and the pathogen spectrum of HFMD has become progressively complex. Although CVB4 is not frequently associated with HFMD outbreaks, it should be focused on as a matter of public health. However, in mainland of China, surveillance for HFMD is mostly focused on EV-A71 and CVA16; therefore, information on the pathogenic role of other EVs, their geographic distribution, and epidemiological profiles is still lacking.

A national or regional EV surveillance system is an essential and effective tool in identifying emerging and re-emerging EV outbreaks and has the potential to inform public health interventions. Therefore, continued surveillance of EVs circulating in China should not only focus only on EV-A71 and CVA16, but also be more comprehensive and include other EV serotypes. EV surveillance should be enhanced to monitor the emergence of new genetic lineages of EV in areas that are often associated with large-scale outbreaks. In addition, continued monitoring of EVs by clinical surveillance and genetic characterization should be encouraged.

To summarize, five CVB4 genotypes are known to have circulated in the world to date, based on entire *VP1* region sequence variation, and all the Chinese CVB4 isolates belong to a new identified genotype (Genotype V), which was identified only in mainland of China since 2007. Intertypic recombination occurred in the Chinese CVB4 strains with novel unknown serotype *EV-B* donor sequences. Chinese CVB4 may be virulent due to the presence of a virulent residue at position 129 of VP1, and 1 strain also has a virulent residue at position 16 of VP4.
